# Matriptase Autoactivation Is Tightly Regulated by the Cellular Chemical Environments

**DOI:** 10.1371/journal.pone.0093899

**Published:** 2014-04-04

**Authors:** Jehng-Kang Wang, I-Jou Teng, Ting-Jen Lo, Sean Moore, Yee Hui Yeo, Yun-Chung Teng, Malvika Kaul, Chiann-Chyi Chen, Annie Hong Zuo, Fen-Pai Chou, Xiaoyu Yang, I-Chu Tseng, Michael D. Johnson, Chen-Yong Lin

**Affiliations:** 1 Department of Biochemistry, National Defense Medical Center, Taipei, Taiwan; 2 Department of Medicine, National Defense Medical Center, Taipei, Taiwan; 3 Geenebaum Cancer Center, University of Maryland, Baltimore, Maryland, United States of America; 4 Department of Biomedical Engineering National Yang Ming University, Taipei, Taiwan; 5 Department of Pharmacology, Rutgers Robert Wood Johnson Medical School, Piscataway, New Jersey, United States of America; 6 Department of Oncology, Lombardi Comprehensive Cancer Center, Georgetown University, Washington DC, United States of America; Lady Davis Institute for Medical Research/McGill University, Canada

## Abstract

The ability of cells to rapidly detect and react to alterations in their chemical environment, such as pH, ionic strength and redox potential, is essential for cell function and survival. We present here evidence that cells can respond to such environmental alterations by rapid induction of matriptase autoactivation. Specifically, we show that matriptase autoactivation can occur spontaneously at physiological pH, and is significantly enhanced by acidic pH, both in a cell-free system and in living cells. The acid-accelerated autoactivation can be attenuated by chloride, a property that may be part of a safety mechanism to prevent unregulated matriptase autoactivation. Additionally, the thio-redox balance of the environment also modulates matriptase autoactivation. Using the cell-free system, we show that matriptase autoactivation is suppressed by cytosolic reductive factors, with this cytosolic suppression being reverted by the addition of oxidizing agents. In living cells, we observed rapid induction of matriptase autoactivation upon exposure to toxic metal ions known to induce oxidative stress, including CoCl_2_ and CdCl_2_. The metal-induced matriptase autoactivation is suppressed by N-acetylcysteine, supporting the putative role of altered cellular redox state in metal induced matriptase autoactivation. Furthermore, matriptase knockdown rendered cells more susceptible to CdCl_2_-induced cell death compared to control cells. This observation implies that the metal-induced matriptase autoactivation confers cells with the ability to survive exposure to toxic metals and/or oxidative stress. Our results suggest that matriptase can act as a cellular sensor of the chemical environment of the cell that allows the cell to respond to and protect itself from changes in the chemical milieu.

## Introduction

Both the intracellular and extracellular chemical environments of a cell play important roles in physiological and pathological processes. To maintain an optimal chemical environment, cells have developed complex mechanisms to monitor and regulate factors such as pH, the concentrations of specific ions, and redox potentials that comprise the chemical milieu of these environments. The secretory pathway maintains a gradient of decreasing pH from near neutrality in the endoplasmic reticulum (ER) (pH 7.2), to mildly acidic in the Golgi (pH 6.7–6.0), to even more acidic within the secretory granules (pH 5.7–5.2) [1]–[4]. This pH gradient is essential both for proper protein sorting and processing [5], as well as for the regulation of enzyme activity [6]. For instance, the activities of proprotein convertases involved in the proteolytic maturation of prohormones are regulated by the pH gradient in the secretory pathway [6]. Another example highlighting the importance of pH in pathophysiological processes is the acidic extracellular environment of solid tumors. The pH of the interstitial fluid in most solid tumors is mildly acidic (6.5), with this value being as low as 5.8 in some cases [7]. This is often the result of tumor hypoxia and is thought to contribute to cancer progression [8]. An example of the importance of extracellular pH and ion concentrations in normal cellular function is the critical pH and calcium gradient required for proper epidermal differentiation and the skin barrier function of the epidermis [9].

Ion channels and G protein-coupled receptors are the two major molecular mechanisms responsible for maintenance of the chemical environment of cells [10]. These membrane proteins perform this function by virtue of being regulated by changes in the chemical environment and acting to counteract those changes. Another class of proteins that can sense and react to the chemical environment is sometimes overlooked in this context: proteases. Proteolytic activity can be regulated by the cellular chemical environment in various ways. For example, an acidic environment can activate several proteases, such as the pro-protein convertases in secretory vesicles, cathepsins in lysosomes, and pepsinogen in the stomach [6], [11], [12].

There is growing evidence that the activity of the type 2 transmembrane serine protease, matriptase, is tightly regulated by the cellular chemical environment [13]. Matriptase, like most proteases, is synthesized as a zymogen and only attains its full enzymatic activity after cleavage at the canonical activation motif. The process of converting matriptase zymogen to the active enzyme is carried out by autoactivation in which interactions between two matriptase zymogen molecules are thought to be responsible for the cleavage of the activation motif via the intrinsic activity of matriptase zymogen [14], [15]. The autoactivation of matriptase was initially suggested after spontaneous activation was observed during the process of refolding of recombinant matriptase serine protease domain [16]. The inability of matriptase mutants with substitutions of the amino acids of the active site triad provided additional evidence for an autoactivation mechanism for the conversion of matriptase zymogen to the active enzyme [14]. Matriptase autoactivation is also dependent on the non-catalytic domains of the enzyme, and posttranslational modifications such as matriptase N-terminal processing and N-linked glycosylation [14]. Interestingly, the G827R matriptase mutation identified in patients with autosomal recessive ichthyosis and hypotrichosis, prevents matriptase from undergoing activation, suggesting that dysregulation of matriptase activation can alter physiological processes and contribute to disease development [17]–[19]. The matriptase autoactivation hypothesis is also supported by the discovery of a matriptase homodimer form recently identified as an intermediate during the process of matriptase activation [15].

The potential role of the chemical environment in the regulation of matriptase activation was initially suggested by the observation that matriptase activation was robustly induced when cells were exposed to a mildly acidic buffer [13], [20]. Acidification appears to significantly enhance the intrinsic activity of the matriptase zymogen and consequently enhances matriptase autoactivation [21]. Matriptase is unique among proteases activated by an acidic pH, the majority of which are secreted or lysosomal proteases, whereas matriptase is anchored onto the surface of cells [22], [23]. Furthermore, matriptase activation can be induced to occur rapidly within minutes by changes in pH [13], [20]. These characteristics of matriptase are similar to ion channels and G protein-coupled receptors in term of location and timing, and suggest that matriptase may act as a sensor that can detect changes in the cellular chemical environment.

In the current study, we set out to systematically analyze the impact of the major chemical components of the extracellular environment on matriptase activation. Our results reveal that matriptase zymogen activation is an autonomous process that can proceed spontaneously at physiological pH and that is accelerated by an acidic pH both in a cell-free setting as well as in living cells. Matriptase zymogen activation is also affected by chloride ion concentration and thio-redox state. Given the rapid induction within minutes of acidification and increased thio-oxidative potential, our current study suggests that the cells could utilize matriptase to sense and respond to alterations in the cellular chemical milieu.

## Materials and Methods

### Cell cultures

The human mammary epithelial cells 184 A1N4 (a gift from M. R. Stampfer, UC Berkeley) [24] were maintained in a modified Improved Minimum Essential Medium (IMEM) supplemented with 0.5% fetal bovine serum, 5 μg/ml recombinant human insulin (rh-insulin), 5 μg/ml hydrocortisone, and 10 ng/ml recombinant human epidermal growth factor (rhEGF). HaCaT human keratinocytes (CLS Cell Lines Service GmbH, Eppelheim Germany) were maintained in Dulbecco's Modified Eagle Medium (DMEM) supplemented with 10% heat-inactivated fetal bovine serum. All cell lines were incubated at 37°C in a humidified atmosphere with 5% CO_2_.

### Western blotting

Cells or the insoluble fractions of cell homogenates were lysed in a buffer consisting of 1% Triton X-100 and 1 mM 5,5′-Dithio-bis-(2-Nitrobenzoic Acid) (DTNB) in phosphate buffered saline (PBS). The Ellman's reagent DTNB was added to the lysis buffer to prevent cleavage of the disulfide linkage that connects the serine protease and the noncatalytic domains of activated matriptase [25]. Lysate protein concentrations were determined by Bradford protein assay and equal amounts of protein were analyzed by Western blotting (immunoblotting). Protein samples for immunoblotting were diluted in 5X sample buffer without a reducing agent and incubated at room temperature for 5 min. Proteins were resolved by 7.5% SDS-PAGE, transferred to nitrocellulose membranes, and probed with the monoclonal antibodies (mAbs) M24 (anti-matriptase) and M19 (anti-HAI-1) [13], [15], [26]. The binding of the primary antibody was detected using horseradish peroxidase (HRP)-conjugated secondary antibodies, and visualized using the Western Lightening^®^ Chemiluminescence Reagent Plus (Perkin-Elmer, Boston, MA).

### Induction of matriptase activation

Matriptase zymogen activation assays were conducted in both the “cell-free” and whole living cell setting using a similar approach. In the cell-free setting, cell homogenates were collected by scraping the cultured cells from the culture dish in PBS and homogenized using a Dounce homogenizer. Aliquots of the homogenate were centrifuged at 12,000 rpm for 5 minutes using an Eppendorf benchtop centrifuge. The pellets of insoluble cell fragments were re-suspended with buffers of different pH and incubated at room temperature for designated times to allow activation to occur. Matriptase activation was terminated by adding 20% Triton-X 100 to a final concentration of 1%. For induction of matriptase zymogen activation in living cells, the cells were washed with PBS three times in 60 mm dishes and incubated with the activation inducers, such as buffers of different pH or modified IMEM containing metal ions at room temperature for the designated times. The cells were then harvested by scraping in PBS. After centrifugation, the cell pellets were lysed in 1% Triton X-100 and 1 mM DTNB in PBS.

### Assay for cytosolic suppressor for matriptase activation

Cells were homogenized in ice-cooled 150 mM phosphate buffer pH 6.0 with a Dounce homogenizer. The homogenates were centrifuged to separate the insoluble cell fractions from the cytosolic fraction. The cytosolic fraction was dialyzed against 150 mM phosphate buffer pH 6.0 using dialysis tubes (MW cutoff 3,500 Dalton). The insoluble fractions were incubated with the cytosolic fractions (freshly prepared, stored, or dialyzed) at room temperature for 20 min. After the incubation, 20% Triton-X 100 was added to the samples to adjust them to 1% Triton-X 100 to stop further matriptase activation. To test the impact of DTNB, oxidized glutathione (GSSG), or N-ethylmaleimide (NEM) on the cytosolic suppressor, these reagents were added to the cytosolic fractions to the indicated concentration before incubation with the insoluble fractions.

### shRNA knockdown

184 A1N4 cells were transduced with lentiviral vectors expressing either a non-targeting control shRNA sequence (NT) or shRNAs targeting the human matriptase gene (MTP) (Open biosystems, Lafayette, CO). The MTP shRNA targeting sequence was

5′-CCGGCAATGACTTCACCTTCGACTACTCGAGTAGT<@?show=[fo]?>CGAAGGTGAA GTCATTGTTTTTG-3′ and has been used and validated by us in previous studies [26]–[28]. Transduced cells were selected with 1 μg/ml puromycin and stable pools of resistant cells were established. Knockdown of matriptase expression was confirmed by Western blot analysis.

### CdCl_2_ toxicity assay

Both matriptase knockdown (MTP KD) and control non-target (NT) 184 A1N4 cells were seeded in 96-well plates at 20,000 cells per well. Sixteen hours later, CdCl_2_ was added to the cells, with final concentrations ranging from 0 to 90 μM. The cells were incubated in CdCl_2_ for 24 hours. Cell survival was determined by estimating the number of cells remaining on the plate using the crystal violet assay. Briefly, the medium was removed from the wells and the cells were fixed and stained with crystal violet by incubation for 10 minutes with 0.52% crystal violet in 25% methanol. The excess stain was removed by washing the plate in deionized water three times after which the plate was dried. Staining intensity was measured by dissolving the crystal violet in 100 mM sodium citrate in 50% ethanol and measuring optical density at 570 nm which is proportional to cell number.

## Results

### Matriptase zymogen activation on isolated cell membrane, is an autonomous process which can be accelerated by mildly acidic conditions

**Figure 1:**
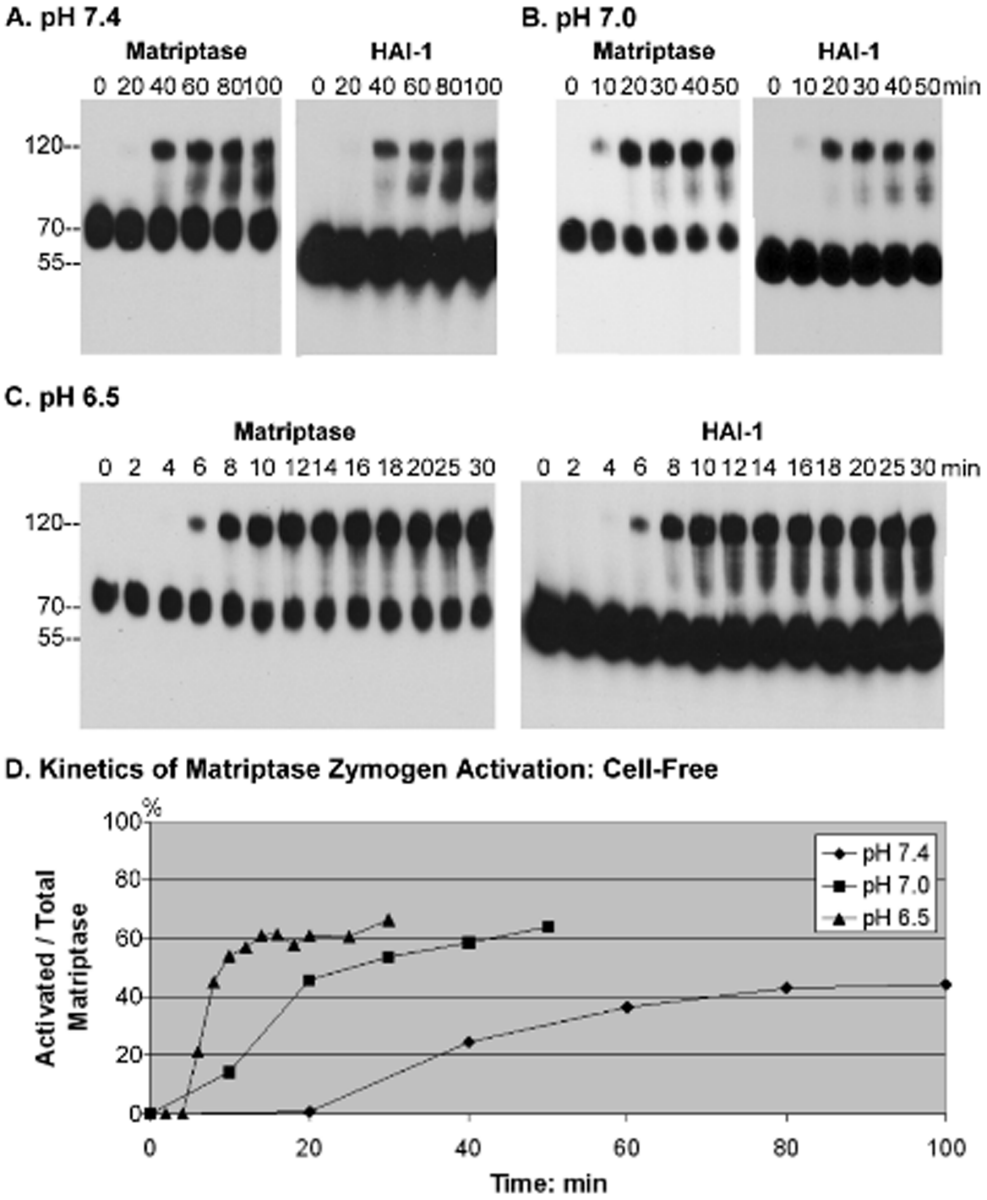
Time and pH-dependence of matriptase zymogen autoactivation in cell-free setting. The insoluble fractions of cell homogenates prepared from 184 A1N4 mammary epithelial cells were exposed to phosphate buffers at pH(A), pH 7.0 (B) or pH 6.5 (C) at room temperature for the indicated time. Lysates were prepared and assayed by immunoblot analyses for matriptase with mAb M24 (which recognizes the 70-kDa matriptase zymogen and 120-kDa activated matriptase-HAI-1 complex) or for HAI-1 with mAb M19 (which recognizes the 55-kDa HAI-1 and 120-kDa activated matriptase-HAI-1 complex). Note that both antibodies also recognize degraded forms of the activated matriptase-HAI-1 complex. (D) Kinetics for induction of matriptase activation in cell-free homogenates. Representative data showing the ratio of activated matriptase relative to total matriptase (activated plus zymogen matriptase) for each pH plotted against time using Image J.

Matriptase is synthesized as a zymogen that undergoes autoactivation to acquire enzymatic activity [14]–[16]. Previous studies have suggested that matriptase autoactivation can be induced within minutes by exposure to mildly acidic conditions in both intact cells and in the insoluble fractions of cell homogenates, the latter representing membrane-bound matriptase in a cell-free setting [20]. Matriptase activation is always followed by the rapid inhibition of active matriptase by binding to its endogenous inhibitor HAI-1 [25], [29]. As a result of these two tightly coupled events, 120-kDa matriptase-HAI-1 complexes are the predominant product of matriptase zymogen activation. When we exposed the insoluble fractions of 184 A1N4 mammary epithelial cells to 150 mM phosphate buffer pH 7.4 at room temperature, activation of matriptase was observed in the fractions within 40 min, with maximal matriptase activation (plateau) being achieved at around 60 minutes (Fig. 1A left panel). The activation of matriptase as indicated by the formation of the matriptase-HAI-1 complex was also detectable using the HAI-1 mAb (Fig. 1A right panel). Anchorage at the cell membrane is required for matriptase activation, as release of matriptase from in the insoluble fraction by addition of the non-ionic detergent Tx-100 (1%) completely abolished matriptase activation [20]. These data suggest that matriptase autoactivation is a biochemical event that can spontaneously occur on the cell membrane at physiological pH in the cell-free setting.

We sought to examine the impact of a lower pH environment on the activation of matriptase in the insoluble fractions by incubating the homogenates with buffers of decreasing pH. When the insoluble fractions were exposed to phosphate buffer at pH 7.0, it took approximately 10 min for the onset of matriptase activation, and about 20 min to reach the maximal matriptase activation plateau (Fig. 1B). When the pH of the incubation buffer was further reduced to 6.5, it took approximately 6 min for the onset of matriptase activation, and about 10 min to reach maximal matriptase activation (Fig. 1C). In addition, exposure to buffers with decreasing pH increased the fraction of the matriptase that was activated relative to total matriptase in the insoluble fraction when maximal matriptase activation was achieved. We used the ratio of the amount of 120-kDa matriptase-HAI-1complex in the sample (which represents the activated fraction of matriptase) to the total matriptase in the sample (determined by sum of the amount of residual 70-kDa matriptase zymogen and the amount of matriptase-HAI-1 complexes) to determine the maximal activation as a percentage under the different conditions. At pH 7.4, only 40% of the matriptase is in its activated form, with this percentage increasing to 60% at pH 7.0, and 60% at pH 6.5 (Fig. 1D). Taken together, these data reveal interesting biochemical features of matriptase zymogen activation, in that as long as matriptase is anchored on the cell membrane, zymogen activation can occur autonomously at physiological pH and that the rate and final extent of matriptase activation is increased at lower pH.

### Exposure to an acidic environment accelerates matriptase zymogen activation in live cells

**Figure 2:**
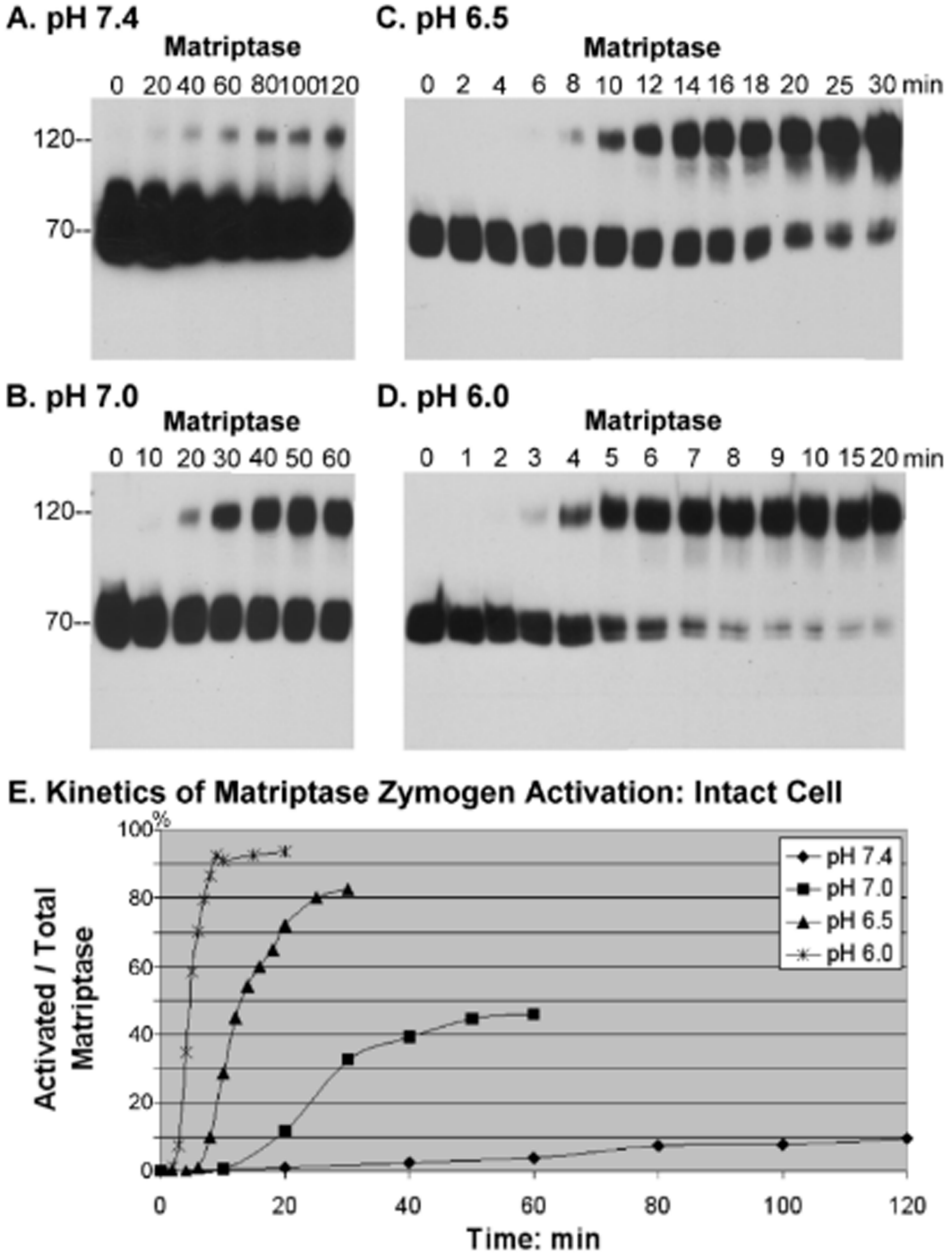
Time and pH-dependence of matriptase zymogen autoactivation in living cells. 184 A1N4 mammary epithelial cells were exposed to phosphate buffers at pH(A), pH 7.0 (B), pH 6.5 (C) or pH 6.0 (D) at room temperature for the indicated time. Cell lysates were prepared and assayed by immunoblot analyses for matriptase using matriptase mAb M24. Matriptase zymogen was detected as a 70-kDa species and activated matriptase was detected as 120-kDa complex with HAI-1. (E) Kinetics of induction of matriptase activation in living cells. The ratio of activated matriptase relative to total matriptase (activated plus zymogen matriptase) for each pH was plotted against time using Image J.

We next sought to investigate whether spontaneous and/or acid-accelerated matriptase activation observed *in vitro* occurs in intact cells. To this end, 184 A1N4 mammary epithelial cells were exposed to 150 mM phosphate buffer at pH 7.4, 7.0, 6.5 or 6.0, and the level of matriptase activation was assessed at various time points by Western blotting (Fig. 2). When the cells were exposed to the buffer at pH 7.4, it took approximately 40–60 min for matriptase activation to begin, with a maximal activation plateau reached at about 80 min (Fig. 2A). When the cells were exposed to buffer at pH 7.0, activation of matriptase was observable within 20 min incubation and reached the plateau after about 30–40 min (Fig. 2B). The acceleration of matriptase zymogen activation was even more pronounced in more acid conditions. At pH 6.5 it took less than 10 min for the onset of matriptase activation, and around 20 min to reach the plateau (Fig. 2C); while at pH 6.0, the onset of matriptase activation occurred at around 3–4 min and the plateau was reached in less than 10 min (Fig. 2D). In addition to the shortened time required for the onset of matriptase activation and to achieve the activation plateau, the percent of matriptase converted to the active form at the plateau phase was also significantly increased by exposure to lower pHs. Over 90% of the matriptase zymogen is converted into matriptase-HAI-1 complex at pH 6.0, whereas at pH 6.5, this percentage was decreased to 80%, at pH 7.0 it was 45% and at pH 7.4, less than 10% of the matriptase zymogen had been converted at the activation plateau (Fig. 2). Taken together, these results indicate that living cells retain the acid-accelerated matriptase zymogen activation observed in the cell-free system.

### Sodium chloride suppresses the induction of matriptase activation by a mildly acidic environment

**Figure 3:**
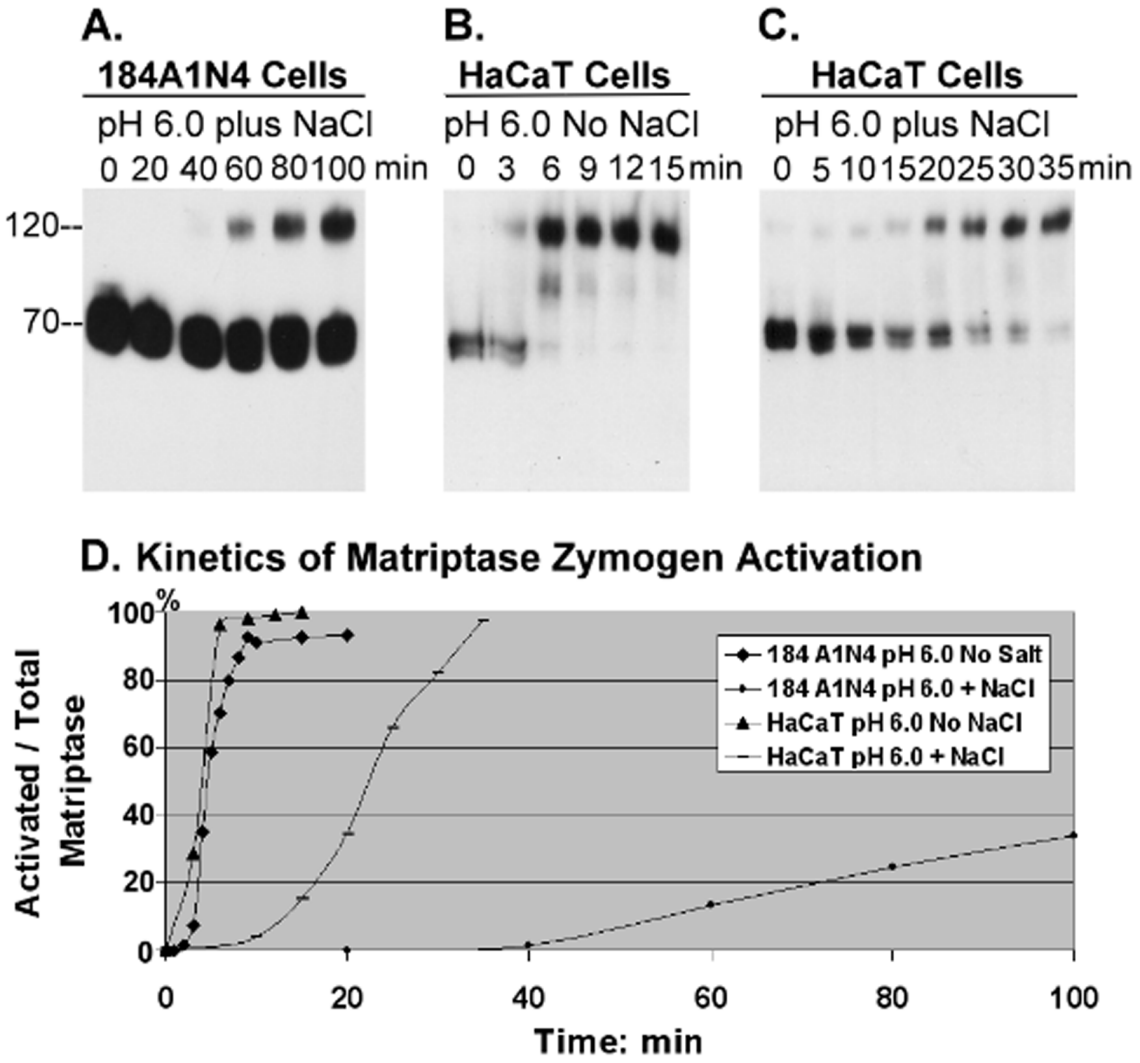
Impact of sodium chloride on matriptase activation. Living 184 A1N4 mammary epithelial cells (A) and HaCaT human keratinocytes (B, C) were exposed to phosphate buffer pH 6.0 at room temperature for the indicated times in the presence (A, C) or absence (B) of 150 mM NaCl. Cell lysates were prepared and assayed by immunoblot analyses for matriptase using mAb M24. Note that the corresponding data for 184 A1N4 mammary epithelial cells exposed to phosphate buffer pH 6.0 in the absence of NaCl is presented in Figure 2D. (D) The impact of NaCl on the kinetics of the induction of matriptase activation in living cells. The ratio of activated matriptase relative to total matriptase (activated plus zymogen matriptase) was plotted against time using Image J.

It is advantageous for cells to be equipped with mechanisms to regulate matriptase activation and particularly the robust acceleration of activation caused by low pH. We have previously described a potential mechanism to regulate acidity-driven matriptase activation in a cell-free system by sodium chloride [20]. Specifically, we showed that the addition of 150 mM sodium chloride to buffer at pH 6.0 suppressed the ability of this pH reduction to activate matriptase in the insoluble fraction [20]. Here we show that living cells share this biochemical feature and demonstrate suppression of matriptase zymogen activation by sodium chloride in an intact cell system. The addition of 150 mM NaCl to the pH 6.0 buffer significantly delayed the onset of activation, as well as reducing the magnitude of matriptase activation in 184 A1N4 mammary epithelial cells (Figure 3A). Activated matriptase was detectable in cells 60 min after exposure to the pH 6.0 buffer containing 150 mM NaCl, with the levels of activation increasing slowly to up to 100 min. This is in stark contrast to the situation in the absence of NaCl, where cells exposed to pH 6.0 buffer exhibited matriptase activation within 3–4 min, with plateau activation reached in less than 10 minutes (Fig. 2D). Furthermore, less than 40% of total matriptase was activated compared to greater than 90% in the absence of NaCl.

We next tested if this is a phenomenon limited to the 184 A1N4 mammary epithelial cells. We tested HaCaT human keratinocyte cells and they demonstrated similar kinetics and magnitude of matriptase activation as the 184 A1N4 mammary epithelial cell line after exposure to NaCl-free buffer at pH 6.0 (Fig. 3B). The addition of 150 mM sodium chloride to the acidic buffer slowed the onset of matriptase activation from 3 min to 15 min, a less dramatic effect than observed for the 184 A1N4 epithelial cells, suggesting that while the effect of NaCl concentration on activation is present in other cell systems, the extent of the attenuation of matriptase activation by NaCl may vary among different cell types. Nevertheless, sodium chloride may play a role in the regulation of the autonomous function of matriptase zymogen activation in matriptase-expressing cells.

### Chloride, but not sodium, ions suppress matriptase zymogen activation

**Figure 4:**
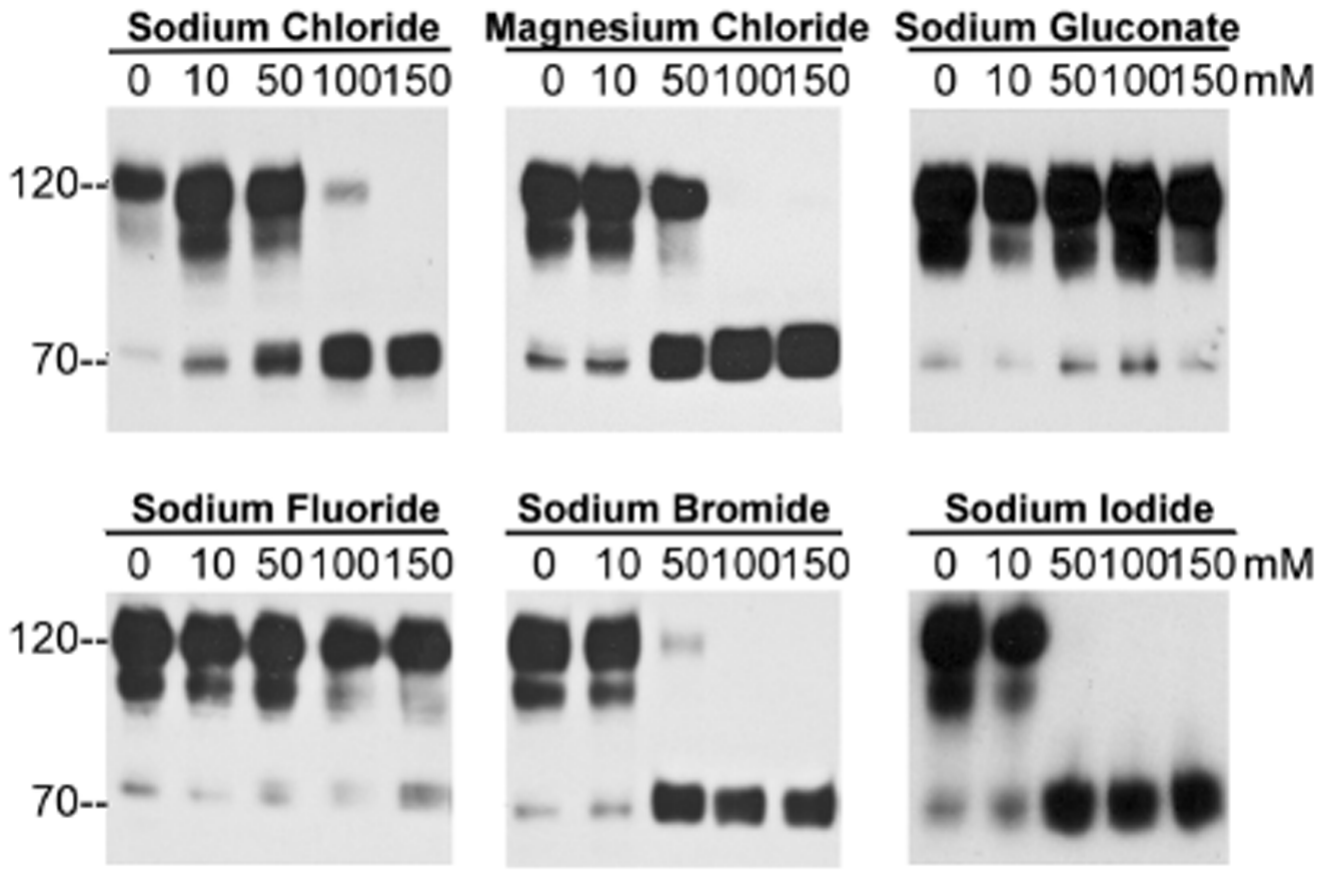
Chloride ions but not sodium ions suppress matriptase zymogen activation. Living 184 A1N4 mammary epithelial cells were exposed to pH

In order to determine which of the ions in sodium chloride, (Na^+^ or Cl^−^) is responsible for mediating the attenuation of matriptase activation, we compared the effects of various salts containing either sodium or chloride ions on matriptase activation in acidic conditions. Both sodium and magnesium chloride inhibit matriptase activation in a dose-dependent manner (Fig. 4, upper panels), whereas sodium gluconate had no effect on matriptase activation, even when used at a concentration of 150 mM (Fig. 4, upper panel). These data indicate that it is the chloride ion, and not the sodium ion, that modulates matriptase activation. In addition to chloride, we discovered that bromide and iodide ions, but not fluoride ions, also inhibited acid-induced matriptase activation (Fig. 4, lower panels). Interestingly, bromide and iodide ions were more potent than the chloride ions in suppressing matriptase activation, suggesting that halogen ions in general can inhibit matriptase activation and that the size of the halide ion may play a role in affecting matriptase activation.

### Cytosolic reductive potential suppresses spontaneous matriptase autoactivation

**Figure 5:**
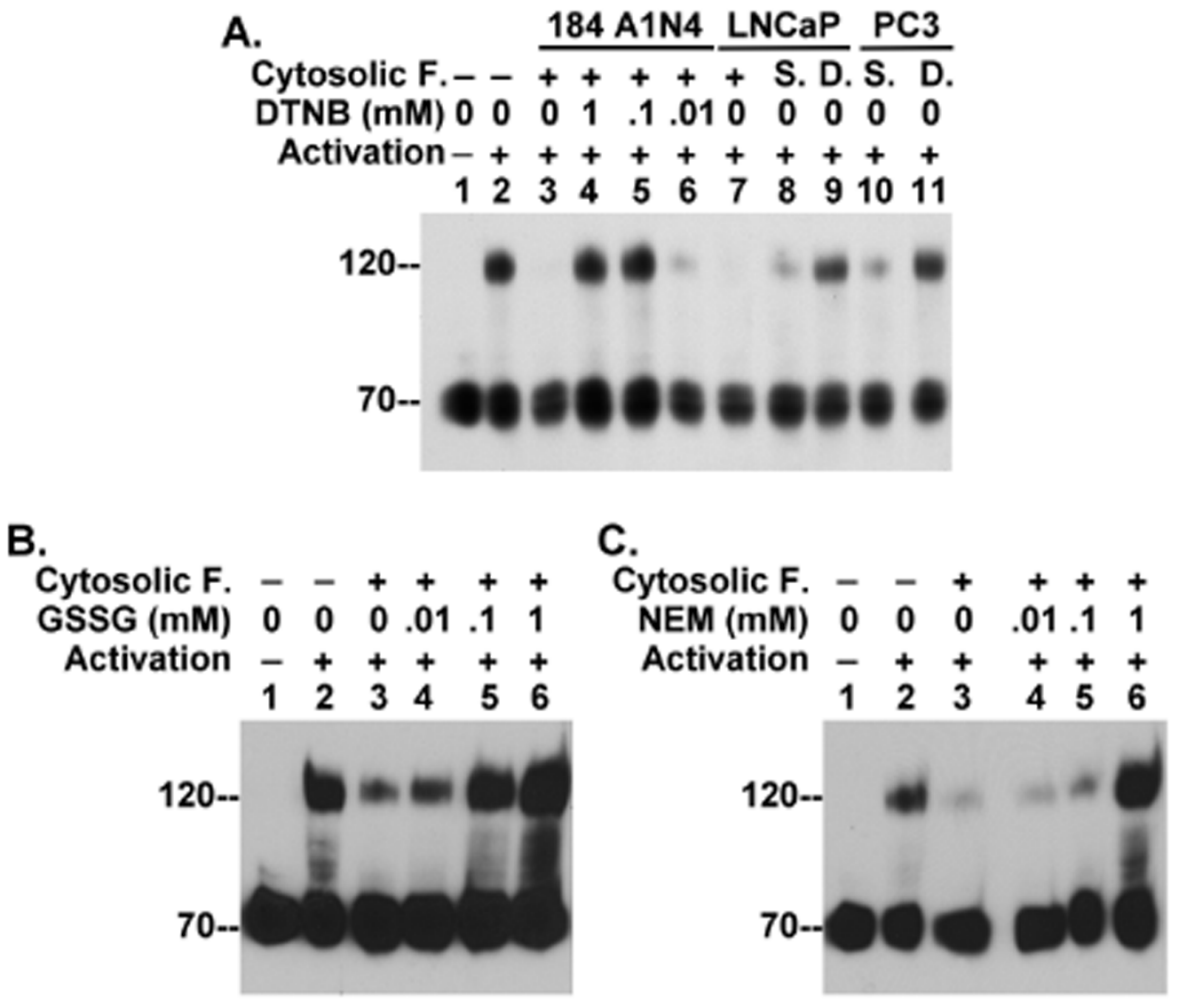
Impact of thio-redox state on matriptase activation. 184 A1N4 cells were homogenized in phosphate buffer pH(*A*) The insoluble fraction was re-suspended and incubated in phosphate buffer pH 6.0 for 20 min either on ice as a negative control (lane 1) or at room temperature as a positive control (lane 2). The insoluble fraction was also mixed with the cytosolic fraction alone (lane 3) or in the presence of various concentrations of DTNB (lanes 4, 5, and 6). Cytosolic fractions were also prepared from LNCaP and PC3 prostate cancer cells and either dialyzed or stored at 4°C for three days. The insoluble fraction prepared from 184 A1N4 cells, was incubated with the untreated, stored (S.), or dialyzed (D.) cytosolic fraction from LNCaP human prostate cells (lanes 7, 8, and 9) and PC3 prostate cancer cells (lanes 10 and 11). (*B* and *C*) The insoluble fraction of 184 A1N4 cells was incubated in phosphate buffer pH 6.0 for 20 min either on ice as a negative control (lane 1) or at room temperature as a positive control (lane 2). The insoluble fraction was also incubated with the cytosolic fraction alone (lane 3) or in the presence of increasing concentrations of oxidized glutathione GSSG (*B*, lanes 4, 5, and 6) or NEM (*C*, lanes 4, 5, and 6). Lysates were prepared from all incubation conditions and assayed by immunoblot analyses for matriptase activation.

The cytosolic concentration of chloride ion is around 5 μM, much lower than the chloride concentration required to impact matriptase activation *in vitro*. We hypothesized, therefore, that there may be other cytosolic mechanism(s) to prevent premature intracellular matriptase zymogen activation. As described above (Fig. 1), in the cell-free system using 184 A1N4 mammary epithelial cell homogenates, matriptase undergoes spontaneous activation in the insoluble fraction only when the insoluble fraction is separated from the cytosolic fraction. We, therefore, tested whether the cytosolic fraction contains a suppressor of matriptase activation. When the cytosolic fraction is added back to the insoluble fraction the activation of matriptase was significantly suppressed (Fig. 5A, lane 3). Furthermore, we found that similar suppression of matriptase activation in the insoluble fraction prepared from 184 A1N4 cells could be suppressed by adding the cytosolic fraction from a variety of differing cell types such as the prostate cancer cells LNCaP (Fig. 5A, lanes 7 and 8) and PC3 (Fig. 5A, lane 10). This suggests that the ability of the cytosolic fraction to suppress activity is conserved among these varied cell types. Characterization of the suppressive activity of the cytosol showed that it was stable when stored for up to three days (Fig. 5A, lanes 8 and 10) but was lost when the cytosol was dialyzed using dialysis tube with molecular weight cutoff of 3,500 D (Fig. 5A, lanes 9 and 11), suggesting that the suppressive activity might depend on one or more small molecules within the cytosol. Significantly, the suppressive activity was neutralized in a dose-dependent manner by Ellman's reagent, 5,5′-dithiobis-(2-nitrobenzoic acid) (DTNB), which oxidizes sulfhydryl groups (Fig. 5A lanes 4, 5, and 6). Similarly, the cytosolic suppressive activity was also neutralized in a dose-dependent manner by the oxidized form of glutathione (GSSG) (Fig. 5B), as well as by N-ethylmaleimide (NEM), which can alkylate sulfhydryl groups (Fig. 5C). These data collectively suggest that the thio-redox state of the cytosolic fraction can impact matriptase zymogen activation, with a more oxidative state favoring matriptase activation while a more reductive state suppressing matriptase activation.

### Impact of transition metals and oxidative stress on matriptase zymogen activation

**Figure 6:**
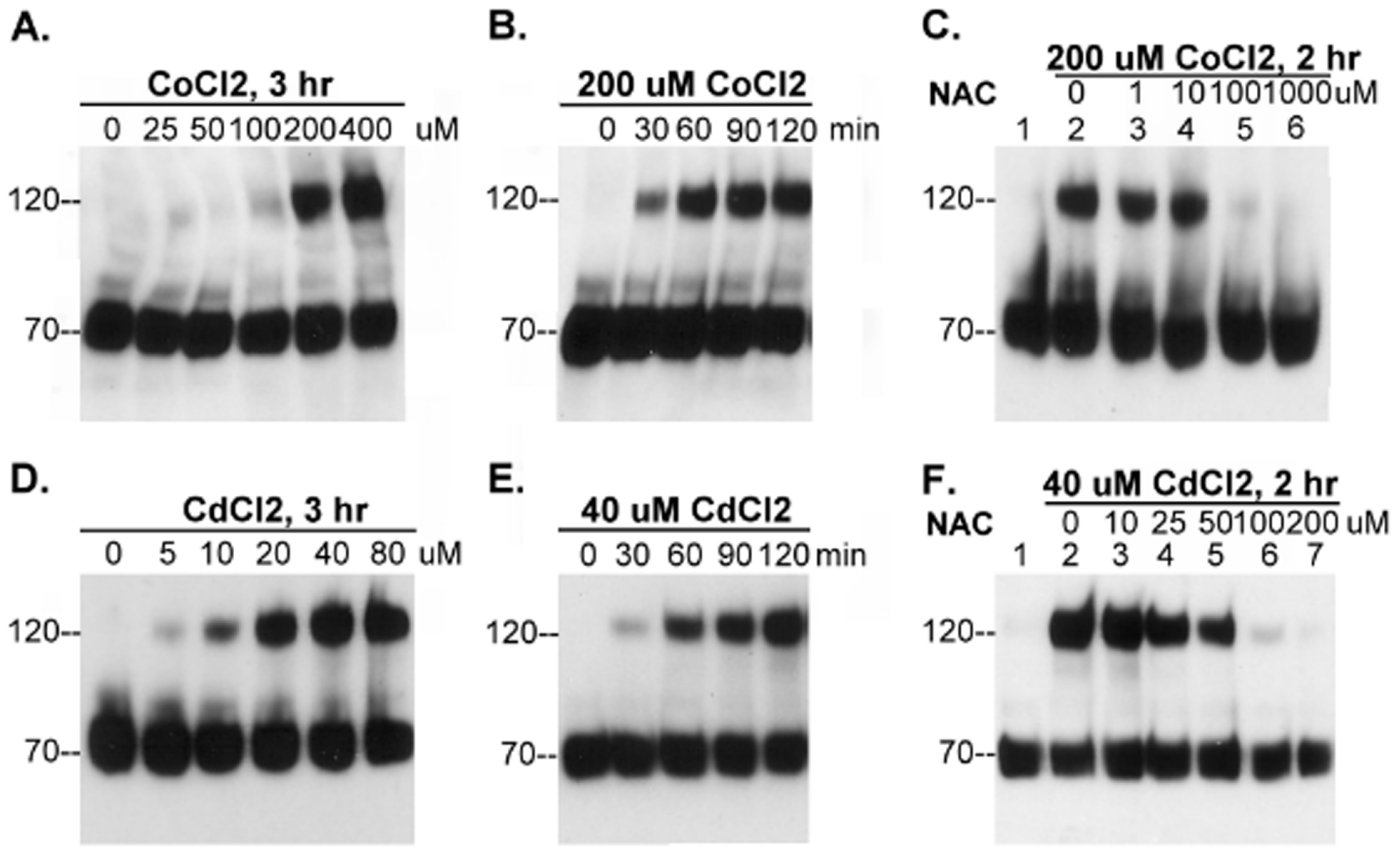
Matriptase zymogen activation can be induced by metal ions. (*A* and *D*) 184 A1N4 cells were incubated with increasing concentrations of CoCl_2_ (*A*) and CdCl_2_ (*D*), as indicated, at 37°C for 3 hrs in a CO_2_ incubator. (*B* and *E*) 184 A1N4 cells were incubated with 200 μM CoCl_2_ (*B*) or 40 μM CdCl_2_ (*E*) at 37°C in a CO_2_ incubator for indicated times. (*C* and *F*) 184 A1N4 cells were incubated with 200 μM CoCl_2_ (C) or 40 μM CdCl_2_ (F) in the presence of increasing concentrations of N-acetylcysteine (NAC), as indicated, at 37°C in a CO_2_ incubator for 2 hrs. Cell lysates were prepared and assayed by immunoblot analyses for matriptase activation using mAb M24 as before.

The thio-redox state-dependence of matriptase zymogen activation that we observed in the cell-free setting prompted us to determine if this biochemical effect is operant in living cells. It has previously been shown that transition metals cause oxidative stress to cells through the production of reactive oxygen species (ROS) and/or depletion of cellular sulfhydryl reserves [30]. As an initial test, we investigated whether matriptase activation can be induced by cobalt chloride (CoCl_2_), which is known to induce rapid ROS production [31] and is widely used as hypoxia mimetic agent. We added various concentrations of the metal ranging from 0 to 400 μM to the basal media in which we cultured 184 A1N4 cells for 3 hours. Exposing the cells to CoCl_2_ induced cellular matriptase activation in a dose-dependent manner (Fig. 6A). We next did a time course study in which we exposed the cells to 200 μM CoCl_2_ and examined matriptase activation in the cells every 30 min for up to 2 hours (Figure 6B). We found that cobalt rapidly induced matriptase activation as indicated by the appearance of the 120-kDa matriptase-HAI-1 complex within 30 min of exposure of the cells to CoCl_2_ (Figure 6B). Furthermore, we found that the antioxidant N-acetyl cysteine (NAC) inhibited CoCl_2_-induced matriptase activation in a dose-dependent manner (Fig. 6C).

To determine if the impact of CoCl_2_ on matriptase activation was a phenomenon limited to cobalt, we also tested other transition metals for their ability to activate cellular matriptase. We found that cadmium chloride (CdCl_2_) was also able to induce matriptase activation and was more potent in this respect than CoCl2, producing a similar level of activation at lower concentrations (Fig. 6D). The kinetics of induction was, however, similar for both compounds (Fig. 6E). As with CoCl_2_, CdCl_2_-mediated matriptase activation was inhibited by NAC in a dose-dependent manner (Fig. 6F). Collectively, these data suggest that matriptase may serve as an early cellular response to oxidative stress induced by metal ions through a thio-redox-regulated zymogen activation process.

### Matriptase expression renders cells more resistant to the toxic effects of CdCl_2_

**Figure 7:**
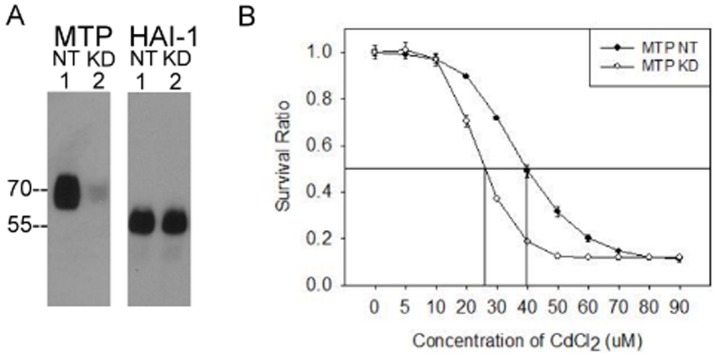
Knockdown of matriptase expression increases susceptibility to CdCl_2_ toxicity. (*A*) Evaluation of matriptase and HAI-1 expression in matriptase knockdown (KD) and non-target (NT) control cells using immunoblot analyses of cell lysates to detect total matriptase (MTP) or HAI-1 (HAI-1). (*B*) Survival rates of matriptase knockdown cells (open circles) in comparison to non-target (NT) control cells (closed circles). Each cell pool was exposed to various concentrations of CdCl_2_, and survival ratios determined by crystal violet staining assay. The results from one representative experiment out of four replicates are shown.

CdCl_2_ is a harmful industrial pollutant known to cause cell death at high concentrations. Oxidative stress is believed to be the primary mediator of toxicity caused by this compound [30], [32]. The rapid and robust induction of matriptase activation by CdCl_2_ suggested the possibility that matriptase might play a role in a survival mechanism induced by cells in response to alterations in cellular chemical environments. To test this hypothesis we generated matriptase knockdown (MTP KD) 184 A1N4 cells using a matriptase targeting shRNA construct, as well as control non-targeted (NT) 184 A1N4 cells using a nonspecific shRNA construct. We verified by immunoblot assay that matriptase protein levels in the MTP KD cells were indeed significantly lower than in the control NT cells (Fig. 7A). We then examined the survival of the cells after exposure to increasing concentrations of CdCl_2_ (0 to 90 μM) for 24 hr. Representative results from one of our experiments are shown in Figure 7B. The data in Figure 7B reveal that the median lethal dose (LD50) of CdCl_2_ for the MTP KD cells was 26 μM, compared to 39 uM for the NT cells. Four independent experiments were conducted, and the average LD50 for MTP KD cells was 23±2 μM; while the average LD50 for the control NT cells 32±5 μM; t-Test, ρ = 0.011). In other words, the MTP KD cells were almost 40% more susceptible to CdCl_2_ than the control NT cells.

## Discussion

In the current study, we show that pH, thio-redox state and chloride ion concentration impact the ability of matriptase to undergo autoactivation, both in a cell free system and in living cells. Interestingly, all three of these factors are considered to be major indicators of the cellular chemical environment that are altered by numerous biological processes. As changes in these cellular indicators also impact matriptase activity, we propose that matriptase may function as an interface between cells and the surrounding chemical environment. This unusual feature could confer matriptase-expressing cells with the ability to sense, respond to, and survive in unfavorable environments such as in the presence of toxic metals.

While living cells resemble the insoluble fraction in the cell-free system with respect to acid-accelerated matriptase zymogen activation, there were some notable differences. At physiological pH, matriptase activation takes longer in living cells than it takes in the cell-free system, and the proportion of total matriptase that undergoes activation is less in living cells at the plateau phase (compare Figs. 1A and 2A). In contrast, at acidic pHs, matriptase activation occurs more rapidly in living cells than in the cell free system and the percent of matriptase activated at the plateau phase is significantly higher (compare Figs. 1C and 2C). The lower level of spontaneous matriptase zymogen activation at physiological pH likely reflects a cellular adaption to ensure that matriptase remains largely in its zymogen state in normal cells in the absence of exogenous stimuli. Furthermore, the increased sensitivity to slight changes in pH would allow living cells to rapidly activate matriptase when encountering pH fluctuations in their environment.

The molecular basis for the rapid and robust activation of matriptase in response to pH, thio-redox state, as well as for the inhibitory effect of chloride ion concentration, remains to be elucidated. It has been previously reported that the intrinsic proteolytic activity of the matriptase zymogen is enhanced in a mildly acidic buffer [21]. This enhanced intrinsic activity could explain, at least in part, the mechanism by which exposure to an acidic environment enhances matriptase activation. Likewise, the inhibition of the intrinsic zymogen proteolytic activity by sodium chloride provides a possible explanation for the role of chloride ion in the suppression of matriptase zymogen activation [21]. Attractive though these explanations might seem, however, caution must be used when trying to link the effects of acidity and sodium chloride on the intrinsic proteolytic activity of matriptase zymogen with their roles in the cellular control of matriptase zymogen activation. The effects of low pH and sodium chloride on the intrinsic zymogen activity were determined in solution using recombinant matriptase [21]. The proteolytic activity under those conditions was so low that it took hours for the detection of matriptase zymogen activity. In contrast, as we have shown, the zymogen activation of matriptase can take place effectively within minutes when matriptase is anchored to the cell membrane either in lysed cells or in intact cells. We have found that if matriptase is liberated from the biomembrane by treatment with non-ionic detergents, matriptase activation in a pH 6.0 buffer does not occur after even 120 min incubation (data not shown). In other words, acidic conditions are not sufficient to initiate the autoactivation of endogenous (rather than recombinant) matriptase zymogen when in solution. The physical environment of matriptase molecules when anchored to the cell membrane seems to be critical to the rapid cleavage and activation of matriptase zymogen. This notion is supported by the fact that the rapid and robust induction of matriptase zymogen activation that can be produced by other agents such as sphingosine 1-phosphate and suramin also involve matriptase presented on a bio-membranes (cell-cell junctions and intracellular membrane structures, respectively) [23], [25].

The molecular mechanism underlying the effect of the thio-redox state on matriptase activation is also not completely understood. One hypothesis is that altered redox state might result in the stabilization of conformations of matriptase that favor the activated form. Matriptase contains 20 pairs of disulfide bonds [16], [33], with one of the disulfide bonds linking the serine protease domain of matriptase with its non-catalytic domain. Previous studies have indicated that the disulfide linkage between the two domains is fragile in activated matriptase complexed with HAI-1, and DTNB must be incorporated in the lysis buffer to prevent artifactual cleavage of this bond [25]. The enhanced matriptase activation we observe under oxidizing conditions might reflect stabilization of activated matriptase through stabilization of its disulfide bonds.

The mechanism through which halogen ions inhibit matriptase activation may also be attributed to their potential impact on matriptase conformation. It should be noted that the hierarchy for matriptase activation inhibition by the halides follows the hierarchy of their sizes (iodide>bromide>chloride). These halogen ions serve as electron acceptors when associated in halogen bonds, with the hierarchy of increasing electron accepting potency also being iodide>bromide>chloride [34]. Halogen bonds are stronger than hydrogen bonds and can compete with hydrogen bonds. Therefore, the presence of halogen ions could stabilize conformations of matriptase zymogen (and other cellular proteins) that are unfavorable for matriptase zymogen activation. It should be noted that fluoride is unable to form halogen bonds, consistent with the inability of NaF to inhibit matriptase activation.

The role of matriptase in many biological processes has been revealed through the identity of its substrates, such as hepatocyte growth factor (HGF), urokinase-type plasminogen activator (uPA), protease activated receptor-2 (PAR2), and prostasin. HGF activation appears to contribute to the oncogenic potential of dysregulated matriptase in the development and progression of squamous cell carcinoma [35]. Matriptase-dependent uPA activation greatly accelerates plasmin generation in monocytes [28], [36]. Activation of PAR-2 by localized proteolysis involving matriptase is important for neural tube closure [37]. In addition to these important and diverse biological activities, matriptase activation can also modulate the cellular chemical environment. Matriptase can either directly, or indirectly through the activation of prostasin, enhance the activity of the epithelial sodium channel [38] and the acid-sensing ion channel [39]. This regulatory loop may allow cells to detect and respond to changes in the extracellular chemical environment.

In addition to expression on the plasma membrane, significant amounts of matriptase are located intracellularly, likely in the synthetic pool and the secretory pathway [22]. Although intracellular matriptase may also become activated in response to extracellular acidosis [13], the presence of high levels of HAI-1 in these compartments would be rapidly inhibit the enzyme through the formation of HAI-1 matriptase complexes. Thus it is unlikely that activated intracellular matriptase plays any significant role in the activation and processing of extracellular matriptase substrates, such as HGF and uPA [40]. Instead, the intracellular activated matriptase may have substrates yet to be identified that are also present in the secretory pathway or on the plasma membrane that matriptase can cleave prior to matriptase inhibition by HAI-1. Currently, prostasin is the only known matriptase substrate that can be activated by matriptase in the face of the rapid HAI-1-mediated inhibition of active matriptase [41]. The identification of substrates of these short-lived pools of active matriptase would provide insights to how matriptase activity is transduced, which could further uncover the functional relationship between matriptase and the cellular chemical environment. The identification of one or more matriptase substrates in polarized epithelial cells is of particular interest, since prostasin may be a keratinocyte-selective matriptase substrate [40].

Matriptase autoactivation is dependent on the cellular chemical environment, with changes in the environment resulting in rapid and robust activation. This unique characteristic of matriptase must be tightly regulated since inappropriate matriptase activation could be extremely damaging to cells if it occurred prior to the delivery of the enzyme to the cell surface. En route to the plasma membrane, matriptase zymogen must traffic through the secretory pathway from the ER, to the Golgi to secretory vesicles, each of which has distinct pH and redox conditions. It is possible that cells have evolved several systems to regulate matriptase so that even if conditions that favor matriptase activation are encountered prematurely, a second mechanism to counteract matriptase activation can suppress it. For instance, in the ER, redox conditions are favorable for matriptase autoactivation, however, this may be sufficiently counteracted by the unfavorable neutral pH. Similarly, in the secretory vesicles, the favorable acidic pH might be negated by the unfavorable reductive potential. On the cell surface, the physiological concentration of chloride ions may serve to moderate excessive matriptase activation potentially induced by extracellular acidosis. Furthermore, in most matriptase-expressing cells, HAI-1 is co-expressed with matriptase at considerable molar excess [15]. HAI-1 can, therefore, serve as a contingent mechanism to quickly inhibit undesired active matriptase caused by temporal and/or sudden changes in the cellular microenvironment.

In summary, the results presented in this study suggest that matriptase, a widespread cell surface serine protease, can function as an interface between cells and their local chemical environment. Matriptase undergoes spontaneous autoactivation at a slow rate in a physiological pH environment, and much of this autoactivation can be suppressed by sodium chloride at physiological concentrations. Increased acidity in the cellular environment accelerates matriptase autoactivation, a mechanism that may be important for certain pathological processes such as cancer. Matriptase autoactivation is also under the tight control of the thio-redox state of the cell environment, with matriptase activation favored under oxidative conditions. The reductive milieu of the cytosol could provide a mechanism to prevent premature matriptase zymogen activation while the protease is trafficking through the gradually acidifying secretory pathway en route to plasma membrane. When the redox balance is altered, such as after exposure to transition metals, matriptase is activated, which may facilitate cell survive under such conditions. Through these positive and negative chemical modulators of matriptase zymogen activation, cells can regulate pericellular proteolysis and respond to the very dynamic cellular chemical environment in a timely and tightly-controlled manner.

## References

[1] Kim JH, Johannes L, Goud B, Antony C, Lingwood CA, et al (1998) Noninvasive measurement of the pH of the endoplasmic reticulum at rest and during calcium release. Proc Natl Acad Sci U S A 95: 2997–3002. 9501204 10.1073/pnas.95.6.2997 PMC19683

[2] Seksek O, Biwersi J, Verkman AS (1995) Direct measurement of trans-Golgi pH in living cells and regulation by second messengers. J Biol Chem 270: 4967–4970. 7890600 10.1074/jbc.270.10.4967

[3] Llopis J, McCaffery JM, Miyawaki A, Farquhar MG, Tsien RY (1998) Measurement of cytosolic, mitochondrial, and Golgi pH in single living cells with green fluorescent proteins. Proc Natl Acad Sci U S A 95: 6803–6808. 9618493 10.1073/pnas.95.12.6803 PMC22642

[4] Miesenbock G, De Angelis DA, Rothman JE (1998) Visualizing secretion and synaptic transmission with pH-sensitive green fluorescent proteins. Nature 394: 192–195. 9671304 10.1038/28190

[5] Paroutis P, Touret N, Grinstein S (2004) The pH of the secretory pathway: measurement, determinants, and regulation. Physiology (Bethesda) 19: 207–215. 15304635 10.1152/physiol.00005.2004

[6] Thomas G (2002) Furin at the cutting edge: from protein traffic to embryogenesis and disease. Nat Rev Mol Cell Biol 3: 753–766. 12360192 10.1038/nrm934 PMC1964754

[7] Tannock IF, Rotin D (1989) Acid pH in tumors and its potential for therapeutic exploitation. Cancer Res 49: 4373–4384. 2545340

[8] Gatenby RA, Smallbone K, Maini PK, Rose F, Averill J, et al (2007) Cellular adaptations to hypoxia and acidosis during somatic evolution of breast cancer. Br J Cancer 97: 646–653. 17687336 10.1038/sj.bjc.6603922 PMC2360372

[9] Menon GK, Grayson S, Elias PM (1985) Ionic calcium reservoirs in mammalian epidermis: ultrastructural localization by ion-capture cytochemistry. J Invest Dermatol 84: 508–512. 3998499 10.1111/1523-1747.ep12273485

[10] Glitsch M (2011) Protons and Ca2+: ionic allies in tumor progression? Physiology (Bethesda) 26: 252–265. 21841073 10.1152/physiol.00005.2011

[11] Richter C, Tanaka T, Yada RY (1998) Mechanism of activation of the gastric aspartic proteinases: pepsinogen, progastricsin and prochymosin. Biochem J 335 (Pt3): 481–490. 10.1042/bj3350481 PMC1219805 9794784

[12] McQueney MS, Amegadzie BY, D'Alessio K, Hanning CR, et al (1997) Autocatalytic activation of human cathepsin K. J Biol Chem. 272: 13955–13960. 10.1074/jbc.272.21.13955 9153258

[13] Tseng IC, Xu H, Chou FP, Li G, Vazzano AP, et al (2010) Matriptase activation, an early cellular response to acidosis. J Biol Chem 285: 3261–3270. 19940125 10.1074/jbc.M109.055640 PMC2823413

[14] Oberst MD, Williams CA, Dickson RB, Johnson MD, Lin CY (2003) The activation of matriptase requires its noncatalytic domains, serine protease domain, and its cognate inhibitor. J Biol Chem 278: 26773–26779. 12738778 10.1074/jbc.M304282200

[15] Xu H, Xu Z, Tseng IC, Chou FP, Chen YW, et al (2012) Mechanisms for the control of matriptase activity in the absence of sufficient HAI-1. Am J Physiol Cell Physiol 302: C453–C462. 22031598 10.1152/ajpcell.00344.2011 PMC3328841

[16] Takeuchi T, Shuman MA, Craik CS (1999) Reverse biochemistry: use of macromolecular protease inhibitors to dissect complex biological processes and identify a membrane-type serine protease in epithelial cancer and normal tissue. Proc Natl Acad Sci U S A 96: 11054–11061. 10500122 10.1073/pnas.96.20.11054 PMC34240

[17] Basel-Vanagaite L, Attia R, Ishida-Yamamoto A, Rainshtein L, Ben AD, et al (2007) Autosomal recessive ichthyosis with hypotrichosis caused by a mutation in ST14, encoding type II transmembrane serine protease matriptase. Am J Hum Genet 80: 467–477. 17273967 10.1086/512487 PMC1821100

[18] List K, Currie B, Scharschmidt TC, Szabo R, Shireman J, et al (2007) Autosomal ichthyosis with hypotrichosis syndrome displays low matriptase proteolytic activity and is phenocopied in ST14 hypomorphic mice. J Biol Chem 282: 36714–36723. 17940283 10.1074/jbc.M705521200

[19] Desilets A, Beliveau F, Vandal G, McDuff FO, Lavigne P, et al (2008) Mutation G827R in matriptase causing autosomal recessive ichthyosis with hypotrichosis yields an inactive protease. J Biol Chem 283: 10535–10542. 18263585 10.1074/jbc.M707012200 PMC2447636

[20] Lee MS, Tseng IC, Wang Y, Kiyomiya K, Johnson MD, et al (2007) Autoactivation of matriptase in vitro: requirement for biomembrane and LDL receptor domain. Am J Physiol Cell Physiol 293: C95–C105. 17344310 10.1152/ajpcell.00611.2006

[21] Inouye K, Yasumoto M, Tsuzuki S, Mochida S, Fushiki T (2009) The Optimal Activity of a Pseudozymogen Form of Recombinant Matriptase under the Mildly Acidic pH and Low Ionic Strength Conditions. J Biochem 147: 485–492. 19919953 10.1093/jb/mvp190

[22] Lin CY, Wang JK, Torri J, Dou L, Sang QA, et al (1997) Characterization of a novel, membrane-bound, 80-kDa matrix-degrading protease from human breast cancer cells. Monoclonal antibody production, isolation, and localization. J Biol Chem 272: 9147–9152. 9083044

[23] Hung RJ, Hsu I, Dreiling JL, Lee MJ, Williams CA, et al (2004) Assembly of adherens junctions is required for sphingosine 1-phosphate-induced matriptase accumulation and activation at mammary epithelial cell-cell contacts. Am J Physiol Cell Physiol 286: C1159–C1169. 15075215 10.1152/ajpcell.00400.2003

[24] Stampfer MR, Bartley JC (1985) Induction of transformation and continuous cell lines from normal human mammary epithelial cells after exposure to benzo[a]pyrene. Proc Natl Acad Sci U S A 82: 2394–2398. 3857588 10.1073/pnas.82.8.2394 PMC397564

[25] Lee M-S, Kiyomiya K, Benaud C, Dickson RB, Lin CY (2005) Simultaneous activation and HAI-1-mediated inhibition of matriptase induced at activation foci in immortal human mammary epithelial cells. Am J Physiol Cell Physiol 288: C932–C941. 15590895 10.1152/ajpcell.00497.2004

[26] Xu Z, Chen YW, Battu A, Wilder P, Weber D, et al (2011) Targeting zymogen activation to control the matriptase-prostasin proteolytic cascade. J Med Chem 54: 7567–7578. 21966950 10.1021/jm200920s PMC3214968

[27] Chen YW, Wang JK, Chou FP, Wu BY, Hsiao HC, et al (2014) Matriptase regulates proliferation and early, but not terminal, differentiation of human keratinocytes. J Invest Dermatol 134: 405–14. 23900022 10.1038/jid.2013.320 PMC3925676

[28] Chou FP, Chen YW, Zhao XF, Xu-Monette ZY, Gartenhaus RB, et al (2013) Imbalanced matriptase pericellular proteolysis contributes to the pathogenesis of malignant B-cell lymphomas. Am J Pathol 183: 1306–1317. 24070417 10.1016/j.ajpath.2013.06.024 PMC3791685

[29] Oberst MD, Chen LY, Kiyomiya KI, Williams CA, Lee MS, et al (2005) Hepatocyte growth factor activator inhibitor 1 (HAI-1) regulates activation and expression of matriptase, a membrane-bound serine protease. Am J Physiol Cell Physiol 289: C462–C470. 15800053 10.1152/ajpcell.00076.2005

[30] Ercal N, Gurer-Orhan H, Aykin-Burns N (2001) Toxic metals and oxidative stress part I: mechanisms involved in metal-induced oxidative damage. Curr Top Med Chem 1: 529–539. 11895129 10.2174/1568026013394831

[31] Chandel NS, Maltepe E, Goldwasser E, Mathieu CE, Simon MC, et al (1998) Mitochondrial reactive oxygen species trigger hypoxia-induced transcription. Proc Natl Acad Sci U S A 95: 11715–11720. 9751731 10.1073/pnas.95.20.11715 PMC21706

[32] Ercal N, Aykin-Burns N, Gurer-Orhan H, McDonald JD (2002) Oxidative stress in a phenylketonuria animal model. Free Radic Biol Med 32: 906–911. 11978492 10.1016/s0891-5849(02)00781-5

[33] Lin CY, Anders J, Johnson M, Sang QA, Dickson RB (1999) Molecular cloning of cDNA for matriptase, a matrix-degrading serine protease with trypsin-like activity. J Biol Chem 274: 18231–18236. 10373424 10.1074/jbc.274.26.18231

[34] Politzer P, Lane P, Concha MC, Ma Y, Murray JS (2007) An overview of halogen bonding. J Mol Model 13: 305–311. 17013631 10.1007/s00894-006-0154-7

[35] Szabo R, Rasmussen AL, Moyer AB, Kosa P, Schafer JM, et al (2011) c-Met-induced epithelial carcinogenesis is initiated by the serine protease matriptase. Oncogene 30: 2003–2016. 21217780 10.1038/onc.2010.586 PMC3084339

[36] Kilpatrick LM, Harris RL, Owen KA, Bass R, Ghorayeb C, et al (2006) Initiation of plasminogen activation on the surface of monocytes expressing the type II transmembrane serine protease matriptase. Blood 108: 2616–2623. 16794252 10.1182/blood-2006-02-001073

[37] Camerer E, Barker A, Duong DN, Ganesan R, Kataoka H, et al (2010) Local protease signaling contributes to neural tube closure in the mouse embryo. Dev Cell 18: 25–38. 20152175 10.1016/j.devcel.2009.11.014 PMC2822780

[38] Andreasen D, Vuagniaux G, Fowler-Jaeger N, Hummler E, Rossier BC (2006) Activation of epithelial sodium channels by mouse channel activating proteases (mCAP) expressed in Xenopus oocytes requires catalytic activity of mCAP3 and mCAP2 but not mCAP1. J Am Soc Nephrol 17: 968–976. 16524950 10.1681/ASN.2005060637

[39] Clark EB, Jovov B, Rooj AK, Fuller CM, Benos DJ (2010) Proteolytic cleavage of human acid-sensing ion channel 1 by the serine protease matriptase. J Biol Chem 285: 27130–27143. 20601429 10.1074/jbc.M110.153213 PMC2930712

[40] Chen YW, Xu Z, Baksh AN, Wang JK, Chen CY, et al (2013) Antithrombin Regulates Matriptase Activity Involved in Plasmin Generation, Syndecan Shedding, and HGF Activation in Keratinocytes. PLoS One 8: e62826. 23675430 10.1371/journal.pone.0062826 PMC3652837

[41] Chen YW, Wang JK, Chou FP, Chen CY, Rorke EA, et al (2010) Regulation of the matriptase-prostasin cell surface proteolytic cascade by hepatocyte growth factor activator inhibitor-1 (HAI-1) during epidermal differentiation. J Biol Chem 285: 31755–31762. 20696767 10.1074/jbc.M110.150367 PMC2951247

